# Optimizing the application order under precedent-based decision-making

**DOI:** 10.1073/pnas.2509985122

**Published:** 2025-07-18

**Authors:** Rossella Argenziano, Itzhak Gilboa

**Affiliations:** ^a^Department of Economics, University of Essex, Colchester CO4 3SQ, United Kingdom; ^b^Department of Economics and Decision Sciences, HEC Paris, Jouy-en-Josas 78351, France; ^c^Tiomkin School of Economics, Reichman University, Herzliya 4610101, Israel

**Keywords:** strategic sequencing, approval decisions, precedents, nearest neighbor, foot-in-the-door

## Abstract

We study the decision problem of a Proposer who has a set of applications to submit for approval to an Authority and can choose an order of submission. The Proposer’s utility depends on the Authority’s rulings. The Authority has to be consistent with its past decisions, which we model using the nearest-neighbor criterion. If the Proposer’s utility increases with the set of approved applications, then any greedy strategy is optimal for her: She should submit any application that, given the current history, would be approved. However, if her utility increases with some approvals but decreases with others, the Proposer’s problem becomes significantly more complex. In the single-dimensional case, an optimal strategy can be computed in polynomial time. In the general case, however, finding an optimal strategy is NP-hard. Thus, even in the absence of uncertainty or strategic behavior on the part of the Authority, evaluating the impact of current submissions on future outcomes can be computationally intractable.

In many social and institutional settings, an individual or organization submits multiple applications to an authority, which can accept or reject each one. Examples include the head of an academic department submitting multiple promotion applications to the provost, a civil rights Non-Governmental Organization filing lawsuits on behalf of multiple plaintiffs, and even a child asking a parent’s permission for different activities. We ask: What is the optimal order in which to submit multiple applications, if the deciding authority is committed to making decisions that are consistent with the precedents it sets?

Consistency with precedents is often a natural constraint on the authority’s decision. Within organizations, consistency of decision-making—such as in promotion decisions—is highly desirable because it is a key component of procedural justice, a concept formalized by refs. 1–3 to describe the fairness of allocation processes within an organization. A broad empirical literature, reviewed by refs. 4 and 5 among others, demonstrates that high levels of perceived procedural justice foster trust, commitment, and improved performance. If organizations therefore strive to be consistent in their decisions, managers may act strategically by choosing the order in which they submit employees for promotion, to enhance the chances of their preferred candidates while disadvantaging others.

In common law systems, courts are bound by precedents: The doctrine of stare decisis requires courts to follow earlier judicial decisions when the same points arise again in litigation (6). Judges apply analogical legal reasoning (7) to identify past cases with material similarities to the one at hand, extract a legal principle from such precedents, and apply it to make a decision. This creates path dependence in judicial decisions (8, 9). Various actors, such as social movement campaigns, leverage this path dependence by using a form of strategic litigation known as test-case litigation, filing strategically chosen lawsuits to establish successful precedents that improve the chances of success of future ones.

At the individual level, preference for consistency has been identified as a measurable personality trait that reflects the tendency to align one’s response to incoming stimuli with previous expectations, commitments, and choices (10). This trait, measured by the Preference for Consistency scale, moderates the effectiveness of consistency-based social influence tactics, such as the foot-in-the-door strategy, in which securing compliance with a small request increases the probability of obtaining compliance with a larger request in the future (10, 11).

In this paper, we introduce a simple model to analyze the decision problem faced by a Proposer who selects the order in which applications are submitted for approval to an Authority that adheres to precedent. Each application has m quality attributes, and applications with the highest possible level of all attributes must be approved, while applications with the lowest possible level of all attributes must be rejected. Apart from these extreme cases, the Authority makes a ruling for each new application by mimicking the ruling in the most similar precedent, identified as the nearest neighbor in the attribute space (see ref. 12). Since the number of permutations of applications is large, the Proposer’s optimization problem is nontrivial.

We show that, if the Proposer’s utility increases monotonically with the set of approved applications, an optimal strategy is easy to compute, and it is greedy: If, given the current set of precedents, there are applications that will be approved if submitted, any one of them can be submitted. Thus, a manager who would like to get all her candidates promoted should submit the strongest applications first, as the success of these applications can only favorably influence the decisions regarding the weaker ones. This mirrors the foot-in-the-door tactic, where an easy initial success increases the likelihood of later successes by lowering the implicit standard for approval.

Strategic considerations become more complex if the Proposer’s preferences are not monotone. For example, if a manager prefers that promotion be denied to some candidates, she might face some nontrivial tradeoffs. We prove that, if the quality of an application is summarized by a single attribute, the Proposer’s problem can still be efficiently solved. By contrast, in a multidimensional setting, the optimization problem is NP-hard. Thus, even in a purely deterministic, single-agent decision problem such as the one we study, evaluating the impact of current submissions on future outcomes can be computationally intractable.

## The Model

An application is a vector $x=\left(x^{1},...,x^{m}\right)\in {\left[0,1\right]}^{m}$, where $x^{j}$ is interpreted as a measure of a certain attribute. For $\alpha \in \left[0,1\right]$, we use $\tilde{\alpha }$ to denote the vector $\left(\alpha ,...,\alpha \right)\in {\left[0,1\right]}^{m}$. A ruling is a binary variable $r\in \left\{0,1\right\}$ interpreted as the approval decision made by the Authority. A case is a pair $c=\left(x,r\right)\in {\left[0,1\right]}^{m}\times \left\{0,1\right\}$ and a history, H, is a sequence of cases. We use the notation$\;H_{i,1}\in {\left[0,1\right]}^{m},H_{i,2}\in \left\{0,1\right\}$ to refer to the two components of the i’th case in H. Thus, $H={\left(H_{i}=\left(H_{i,1},H_{i,2}\right)\right)}_{i\leq t}$ (t≥0).

The Proposer has a set of applications that it has to submit to the Authority for approval. We denote it by $\left(x_{1},...,x_{n}\right)$ and use a sequence to make the notation $x_{i}$ well-defined and to allow repeated applications. However, no particular meaning should be attached to the order of $x_{i}$ in the sequence. The Proposer has to choose a permutation of the applications, that is, the order in which applications will be submitted to the Authority. When all applications are submitted and ruled, the ruling combination $\overset{¯}{r}={\left(r_{i}\right)}_{i\leq n}$ is the outcome. The utility that the Proposer derives from ruling combination $\overset{¯}{r}={\left(r_{i}\right)}_{i\leq n}$ is

$$U\left(\overset{¯}{r}\right)=\sum_{i=1}^{n}u\left(x_{i},r_{i}\right),$$

where $u\left(x_{i},r\right)\in R$ for each i≤n and $r\in \left\{0,1\right\}$. The restriction to an additive function is imposed for simplicity. The results hold also for more general functions, subject to computability constraints, as explained below.

We consider a history that has two “paradigmatic cases”: an all-0 application (real or hypothetical), which is ruled as 0, and similarly, an all-1 application, which is ruled as 1. These generate the history $H^{2}=\left(\left(\tilde{0},0\right),\left(\tilde{1},1\right)\right)$. At stage t (t≥3) a history $H={\left(H_{i}=\left(H_{i,1},H_{i,2}\right)\right)}_{i\leq t}$ is given, and the Proposer submits an application x out of $\left(x_{1},...,x_{n}\right)$, which has not been submitted yet ($x\neq H_{i,1}$ for 2<i≤t). The Authority makes a ruling r, which has to be a nearest-neighbor ruling: $r=r_{q}$ for $q=\;\min\left[\arg\;\min_{l\leq t}\left(∥x-x_{l}∥\right)\right]$. That is, we consider the past $x_{l}$’s that minimize the Euclidean distance from the new x. (This can be generalized to a weighted Euclidean distance without affecting the results in the paper.) The argmin is the set of the corresponding indices, viewing $\left(∥x-x_{l}∥\right)$ as a function of l≤t. If there is a single minimizer of the distance, $x_{q}$, we select the ruling $r_{q}$ for the current application x. In case of multiple minimizers, for simplicity we select the one that appears first in the history H, to make sure that the Authority has no decisions to make even in case of ties. History H is then extended to ${\left(\left(H_{i,1},H_{i,2}\right)\right)}_{i\leq t+1}$ with $H_{i+1,1}=x$ and $H_{i+1,2}=r$. A history that is generated in this way is referred to as NN-consistent. That is, $H={\left(H_{i}=\left(H_{i,1},H_{i,2}\right)\right)}_{i\leq t}$ (t≥2) is NN-consistent if, for every 2<i≤t we have $r_{i}=r_{q}$, where $q=\;\min\left[\arg\;\min_{l<i}\left(∥x_{i}-x_{l}∥\right)\right]$. Since the Authority does not have any freedom in choosing the rulings, we may assume that the Proposer selects a permutation of $\left(x_{1},...,x_{n}\right)$ at the outset. Thus, the Proposer has n! pure strategies.

For a permutation $\pi :\left\{1,...,n\right\}\to \left\{1,...,n\right\}$ there exists a unique NN-consistent history of length n+2,

$$H^{\pi }=\left(\left(\tilde{0},0\right),\left(\tilde{1},1\right),\left(x_{\pi \left(1\right)},r_{\pi \left(1\right)}\right),...,\left(x_{\pi \left(n\right)},r_{\pi \left(n\right)}\right)\right).$$

The history $H^{\pi }$ defines a function $f^{\pi }:{\left(x_{i}\right)}_{i\leq n}\to \left\{0,1\right\}$. Let the vector ${\overset{¯}{r}}^{\pi }\equiv {\left(r_{i}^{\pi }\equiv f^{\pi }\left(x_{i}\right)\right)}_{i\leq n}$ be the ruling combination defined by π.

## Results

### Monotone Preferences.

A function U is monotone if $u\left(x_{i},1\right)>u\left(x_{i},0\right)$ for all i.

A Proposer’s strategy is greedy if the following holds: For every history $H={\left(H_{i}=\left(H_{i,1},H_{i,2}\right)\right)}_{i\leq t}$ (t≥2) if there exists x that is not yet in H ($x\neq H_{i,1}$ for 2<i≤t) and will be ruled as 1 ($r_{q}=1$ for $q=\;\min\left[\arg\;\min_{l\leq t}\left(∥x-x_{l}∥\right)\right]$), then such an x is submitted. In other words, a greedy strategy picks applications that will be ruled as 1 before it selects any application that will be ruled as 0.

Proposition 1 *If the Proposer’s preferences are monotone, any greedy strategy is an optimal strategy, and they all result in the same ruling combination* $\overset{¯}{r}$. *Moreover, if* $r_{i}=1$ *for a ruling combination* r *defined by some strategy* π, *then* ${\overset{¯}{r}}_{i}=1$. *Finally, a greedy strategy can be found in polynomial time*.

This proposition captures the idea that if the Proposer wishes to get some applications approved, she should start with those applications that can be approved at present, and proceed from these on. Note that the set of all strategies the Proposer can select from is exponentially large, and the set of all greedy strategies may well be exponentially large as well. One might wonder, then, whether the Proposer’s problem can be optimally solved in polynomial time. The Proposition guarantees that this is indeed the case. Any greedy strategy is optimal, and at least one such strategy can be found in polynomial time. Not only will all these strategies result in the same ruling combination, the latter will include (as r=1) all the applications that can ever be ruled as 1.

We note that the proposition holds true also if the Proposer’s utility U is not additive, as long as it is a function of the rulings (and not their order), and it is monotone with respect to set inclusion (of the 1 rulings).

### General Preferences.

There are cases in which the Proposer’s preferences might not be monotone. For example, a department chair might not like a certain candidate due to the latter’s field of research (or personality). Thus, we can have $u\left(x_{i},1\right)<u\left(x_{i},0\right)$ for some i’s. In this case, we can redefine a “greedy” strategy to be a strategy that picks an application with $u\left(x_{i},1\right)>u\left(x_{i},0\right)$ if there is such an application that will be ruled as 1 (given current history H), and/or an application with $u\left(x_{i},1\right)<u\left(x_{i},0\right)$ if there is such an application that will be ruled as 0. However, such strategies need not be optimal, as the following example illustrates.

Example Let m=1 and n=2. Let $x_{1}=0.4$ and $x_{2}=0.6$ with $u\left(x_{i},r\right)$ given by

$$\begin{array}{c} \begin{array}{ccc} & r=0 & r=1\\ x_{1}=0.4 & 10 & 1\\ x_{2}=0.6 & 0 & 1 \end{array} \end{array}$$

There are only two strategies, corresponding to the permutations $\left(1,2\right)$ and $\left(2,1\right)$. Given the initial history $H^{2}=\left(\left(0,0\right),\left(1,1\right)\right),$ either application will be ruled as desired if (and only if) submitted first. If $x_{1}=0.4$ is submitted first, the rulings are $r_{1}=r_{2}=0$ and U(0,0)=10, while if $x_{2}=0.6$ is submitted first, we have $r_{1}=r_{2}=1$, and U(1,1)=2. Hence, both strategies are greedy, but only the first one is optimal.

Thus, finding an optimal strategy for a Proposer with nonmonotone preferences is a nontrivial task. Given that there are n! possible strategies, one might wonder whether finding an optimal one is a reasonable task to perform. The answer turns out to be positive in the single-dimension case.

Theorem 1 *Assume that* m=1. *There exists a polynomial-time algorithm that, given* ${\left(x_{i}\right)}_{i\leq n}$ *and* u, *finds a permutation* π *that maximizes* $U\left({\overset{¯}{r}}^{\pi }\right)$.

This result would hold for any function U that is monotone in the set of 1-rulings, provided that the value of the function can be computed in polynomial time (in n,m).

By contrast, if the dimensionality of the application is not restricted (so that m is part of the problem’s input), the problem is hard. (See ref. 13, for a similar contrast between the uni- and multidimensional case in a legal context.)

Theorem 2 *Given* m, ${\left(x_{i}\right)}_{i\leq n}$, *and* u, *finding a permutation* π *that maximizes* $U\left({\overset{¯}{r}}^{\pi }\right)$ *is NP-hard*.

This result suggests that, in the general case, the Proposer might not be able to foresee the outcome of her choices. Even though the model is perfectly deterministic, and the Proposer is the only strategic player in the game, the sheer complexity of the problem makes it hard to imagine how applications that are submitted to the Authority at present will affect future ones. Note that, since the theorem holds for an additive U, it certainly holds true for more general functions.

## Discussion

Our model assumes that time does not play a role and that the world is completely deterministic. Clearly, these are highly idealized assumptions. Proposers might be impatient, and authorities’ decisions are subject to uncertainty. Our analysis could be extended to these circumstances. Similarly, one may wish to replace the nearest-neighbor approach with different notions of consistency with precedents, or augment it in various ways. For example, one could allow for biased assessments of attribute distance by the Authority (see ref. 14) or consider a distance that takes into account a hierarchy of precedent (as in ref. 9).

A more fundamental extension of our model would treat both Proposers and Authorities as strategic players, and allow for multiple players of one or both types. In many contexts, multiple Proposers submit applications that may influence each other’s outcomes, while multiple Authorities enable forum shopping. While this paper does not analyze such strategic interactions, it lays the computational groundwork for identifying optimal strategies in these games.

## Supplementary Material

Appendix 01 (PDF)

## Data Availability

There are no data underlying this work.

## References

[1] G. S. Leventhal, “Justice in social relationships” in Contemporary Topics Social Psychology, J. W. Thibaut, J. T. Spence, R. C. Carson, Eds. (General Learning Press, Morristown, NJ, 1976), pp. 211–240.

[2] G. S. Leventhal, “What should be done with equity theory? New approaches to the study of fairness in social relationships” in Social Exchange: Advances in Theory and Research, K. J. Gergen, M. S. Greenberg, R. H. Willis, Eds. (Springer, New York, NY, 1980), pp. 27–55.

[3] G. S. Leventhal, Beyond fairness: A theory of allocation preferences. Justice Soc. interact. **3**, 167 (1980).

[4] Y. Cohen-Charash, P. E. Spector, The role of justice in organizations: A meta-analysis. Organ. Behav. Hum. Decis. Process. **86**, 278–321 (2001).

[5] J. A. Colquitt, D. E. Conlon, M. J. Wesson, C. O. Porter, K. Y. Ng, Justice at the millennium: A meta-analytic review of 25 years of organizational justice research. J. Appl. Psychol. **86**, 425 (2001). 11419803 10.1037/0021-9010.86.3.425

[6] B. Garner, *Black’s Law Dictionary* (Thomson Reuters, 2014).

[7] E. H. Levi, An Introduction to Legal Reasoning (University of Chicago Press, 2013).

[8] O. A. Hathaway, Path dependence in the law: The course and pattern of legal change in a common law system. Iowa L. Rev. **86**, 601 (2000).

[9] J. C. Teitelbaum, Analogical legal reasoning: Theory and evidence. Am. Law Econ. Rev. **17**, 160–191 (2015).

[10] R. B. Cialdini, M. R. Trost, J. T. Newsom, Preference for consistency: The development of a valid measure and the discovery of surprising behavioral implications. J. Pers. Soc. Psychol. **69**, 318 (1995).

[11] J. L. Freedman, S. C. Fraser, Compliance without pressure: The foot-in-the-door technique. J. Pers. Soc. Psychol. **4**, 195 (1966). 5969145 10.1037/h0023552

[12] E. Mackaay, P. Robillard, Predicting judicial decisions: The nearest neighbour rule and visual representation of case patterns. Datenverarb. Recht **3**, 302–331 (1974).

[13] J. C. Teitelbaum, Computational complexity and tort deterrence. J. Leg. Stud. **51**, 249–288 (2022).

[14] D. Teichman, E. Zamir, “Judicial decision-making: A behavioral perspective” in The Oxford Handbook of Behavioral Economics and the Law, E. Zamir, D. Teichman, eds. (Oxford University Press, Oxford, UK, 2014).

