# Supplementary material for: Optimizing the application order under precedent-based decision-making
Source: Proc Natl Acad Sci U S A. 2025 Jul 18;122(29):e2509985122. doi: 10.1073/pnas.2509985122 (PMC12304939; doi:10.1073/pnas.2509985122)
Supplement: Supplementary file 1 — Appendix 01 (PDF) [file pnas.2509985122.sapp.pdf]

# Supporting Information for

## Optimizing Application Order Under Precedent-Based Decision Making

**Rossella Argenziano and Itzhak Gilboa**

**Itzhak Gilboa**
**tzachigilboa@gmail.com**

**This PDF file includes: Proofs**

## Proofs

**Proof of Proposition 1.** Consider a permutation $\pi$ of $\{1,...,n\}$ that does not define a greedy strategy. Let $t \le n$ be the first step, according to $\pi$, that violates the greediness condition: there is a history $H = (H_i = (H_{i,1}, H_{i,2}))_{i \le t}$, and there exists $x$, not appearing in $H$, which would be ruled as $r = 1$, but another application, $x'$, is presented next and is ruled as $r' = 0$. Consider the permutation $\pi^*$ obtained from $\pi$ by moving $x$ just before $x'$ and leaving the other applications intact. Clearly, the first $t$ applications are ruled as before. The new application $x$ is ruled as $r = 1$. For any application that appears in the permutation, if it were ruled as 1 according to $\pi$, it will also be so ruled in $\pi^*$ (by induction over the remainder of $\pi$). Applying this argument inductively, we conclude that there exists a greedy strategy $\pi^{**}$ that obtains a ruling combination $\bar{r}^{**}$ such that $r_i = 1$ implies $r_i^{**} = 1$. In other words, the set of 1-rulings according to the greedy strategy $\pi^{**}$ contains that of the original $\pi$. In particular, $\pi^{**}$ obtains at least as high a value of $U^P$ as does $\pi$.

The above means that, looking for a maximal $U^P$, it suffices to consider the greedy strategies. Yet, it is a priori possible that different greedy strategies would yield different ruling combinations $\bar{r}$'s, and different payoffs. We wish to show that this is not the case. Specifically, we wish to show that for any two permutations $\pi, \pi'$ that define greedy strategies, the ruling combinations are identical. Let $\pi, \pi'$ be such permutations. Consider the first application in $\pi$. If it is ruled as 0, then all applications are closer to 0 than to 1[*] and both $\pi$ and $\pi'$ define the empty set (of 1-rulings). Assume, then, that $x_i$ is the first application in $\pi$, and that it is ruled as 1 (according to $\pi$). In $\pi'$ application $x_i$ may appear later on, but, we argue, it has to be ruled as 1 as well: assume not, so that $x_i$ is ruled as 0 in $\pi'$. Then there exists an application $x_j$, appearing before $x_i$ in $\pi'$, which is ruled as 0 in $\pi'$ (and which is closer to $x_i$ than any other application preceding $x_i$ in $\pi'$). Let $x_l$ be the earliest application in $\pi'$ that is ruled as 0 (irrespective of its distance from $x_i$). At the point that $x_l$ is presented in $\pi'$, it is about to be ruled as 0 whereas $x_i$ would be ruled as 1. This is a contradiction to the greediness of $\pi'$. Thus, the first application that is ruled as 1 in $\pi$ is also ruled as 1 in $\pi'$. We continue the argument inductively: we assume that the first $k$ applications that appear in $\pi$ and are ruled as 1 are also ruled as 1 in $\pi'$, and consider the $(k+1)$-th application if such exists. Assume that this application is $x_i$ and that its preceding nearest-neighbor in $\pi$ is $x_p$. By the induction hypothesis, $x_p$ is ruled as 1 also at $\pi'$. If $x_i$ is ruled as 0 in $\pi'$, there exists an application $x_j$, appearing before $x_i$ in $\pi'$, which is ruled as 0 in $\pi'$. Let $x_l$ be the earliest application in $\pi'$ that is ruled as 0 and consider the point that $x_l$ is presented in $\pi'$. Because $\pi'$ is greedy, and $x_p$ is ruled as 1 in $\pi'$, it has to be the case that $x_p$ precedes $x_l$ in $\pi'$. But then, at this point (that is presented in $\pi'$) presenting $x_i$ would result in a 1 ruling. This means that a greedy $\pi'$ cannot present $x_l$ before $x_i$, a contradiction. We thus conclude that all the applications ruled as 1 in $\pi$ are also ruled as 1 in $\pi'$. The argument being symmetric, we find that any two greedy permutations define the same ruling.

It remains to be noted that finding one such permutation is a polynomial task, and can be performed in $O\left(n^2\right)$ steps: at each stage, we can go over all the applications not yet encountered, and should one of them qualify as a 1-ruling, choose it and continue to the next stage.[†] □

**Proof of Theorem 1.** Assume, wlog, that $x_i \le x_j$ for $1 \le i < j \le n$.

We say that a ruling combination $\bar{r} = (r_i)_{i \le t}$ is monotone if $r_i \le r_j$ whenever $i < j$. Equivalently, $\bar{r}$ is monotone if there exists $\gamma \in (0,1)$ for which [$r_i = 0$ for $x_i$ iff $x_i < \gamma$ (for all $i$)].

We first note that, if a history $H = (c_i = (x_i, r_i))_{i \le t}$ is NN-Consistent, then $\bar{r} = (r_i)_{i \le t}$ is monotone. Let $H = (c_i = (x_i, r_i))_{i \le t}$ with $c_1 = (0,0)$ and $c_2 = (1,1)$. Clearly, $H^2 = (c_1, c_2)$ is monotone. Let $k$ be the minimal index such that $H^k = (c_1, c_2, ..., c_k)$ isn't monotone. Then $(c_1, c_2, ..., c_{k-1})$ is monotone, which means that there exists $\gamma \in (0,1)$ so that, for all $i < k$, $r_i = 0$ if $x_i < \gamma$ and $r_i = 1$ if $x_i > \gamma$. If $x_k \in (\max_{x_i<\gamma} x_i, \min_{x_i>\gamma} x_i)$, then $\bar{r} = (r_i)_{i \le t}$ is also monotone (whether $r_k = 0$ or $r_k = 1$). As this is not the case, we must have $x_k \le \max_{x_i<\gamma} x_i$ or $x_k \ge \min_{x_i>\gamma} x_i$. In the former case the closest neighbors to $x_k$, from above and below are both ruled as 0, and NN-consistency implies that $r_k = 0$, and the symmetric argument applies to the latter case.

There are $(n+1)$ monotone ruling combinations. Specifically, for each $b \in \{0, ..., n\}$ define $\bar{r}^b \equiv \left(r_i^b\right)_{i \le n}$ by [$r_i^b = 0$ for $i \le b$ and $r_i^b = 1$ for $i > b$]. Thus, for each permutation $\pi$ there exists $b = b(\pi)$ such that $\bar{r}^\pi = \bar{r}^b$.

For each $b \in \{0, ..., n\}$, the computation of $U^P\left(\bar{r}^b\right)$ can be done in polynomial time (in $n$) by the additivity assumption. Hence, we focus on the following subproblem: given $b \in \{0, ..., n\}$, can we find a permutation $\pi$ so that $\bar{r}^\pi = \bar{r}^b$? If we can solve this problem in polynomial time, we can also maximize $U^P$ in polynomial time: we only need to compare the feasible ruling combinations (whose number is no more than $(n+1)$) and select a maximizer of $U$ among them. We now turn to prove that there exists a polynomial-time algorithm that solves this subproblem.

Let there be given a $b \in \{0, ..., n\}$. We define a permutation $\pi$ to be $b$-monotone if:

(i) For every $i < j \le b$, $\pi^{-1}(i) < \pi^{-1}(j)$;

(ii) For every $i > j > b$, $\pi^{-1}(i) < \pi^{-1}(j)$.

In other words, $\pi$ is $b$-monotone if all the applications whose indexes are up to $b$ appear in a monotonically increasing order, and all the applications whose indexes are above $b$ appear in a monotonically decreasing order. Intuitively, all the applications that have to be ruled as 0 are presented in order of their proximity to 0, and all those that have to be ruled as 1 – in order of their proximity to 1.

[*] Taking into account the tie-breaking rule we assumed.

[†] One may refine the algorithm to mimic breadth-first-search or a depth-first-search.

**Lemma 1.** *If there exists a permutation $\pi$ such that $\bar{r}^{\pi} = \bar{r}^{b}$, then there also exists a $b$-monotone permutation $\pi$ such that $\bar{r}^{\pi} = \bar{r}^{b}$.*

**Proof of Lemma 1:** Assume that a permutation $\pi$ with $\bar{r}^{\pi} = \bar{r}^{b}$ exists, but that it is not $b$-monotone. Assume first that it fails to satisfy (i). Let $i$ be the minimal index for which there exists $j$, $i < j \leq b$, with $\pi^{-1}(j) < \pi^{-1}(i)$. Let $j$ be the maximal such index for $i$. Thus, $x_j$ is the maximal $x$ that appeared before $i$ and was ruled as $r_j = 0$.

Consider the permutation $\pi'$ that differs from $\pi$ only by moving $i$ to the spot before $j$ and leaving the other indexes unchanged. That is, $\pi'$ is defined by: (1) $\pi'^{-1}(l) = \pi^{-1}(l)$ for all $l$ with $\pi^{-1}(l) < \pi^{-1}(j)$; (2) $\pi'^{-1}(i) = \pi^{-1}(j)$; (3) $\pi'^{-1}(l) = \pi^{-1}(l) + 1$ for all $l$ with $\pi^{-1}(j) \leq \pi^{-1}(l) < \pi^{-1}(i)$; and (4) $\pi'^{-1}(l) = \pi^{-1}(l)$ for all $l$ with $\pi^{-1}(l) > \pi^{-1}(i)$.

We claim that $\bar{r}^{\pi'} = \bar{r}^{\pi}$. We prove the claim inductively, proceeding over (i)-(v) in the given order, as this is the order dictated by $\pi'$.

(1) For $l$ with $\pi^{-1}(l) < \pi^{-1}(j)$, each application is presented after the same history according to $\pi$ and to $\pi'$.

(2) Consider application $x_i$, which is located at spot $\pi'^{-1}(i) = \pi^{-1}(j)$ according to $\pi'$. According to the permutation $\pi$, $x_i$ was ruled to be $r_i = 0$ (as it should be, based on $\bar{r}^{b}$). Could it be ruled as $r_i = 1$ according to $\pi'$? We argue that the answer is negative. When $x_i$ is presented according to $\pi'$, the history is the $\left(\pi^{-1}(j) + 1\right)$-prefix of $H^{\pi}$, which is identical to the $\left(\pi^{-1}(j) + 1\right)$-prefix of $H^{\pi'}$.[‡] Given this history, $x_j$ was ruled as 0. We argue that, given the same history, $x_i (< x_j)$ will also be ruled as 0. Indeed, the maximality of $x_j$ guarantees that there are 0 rulings only to its left (i.e., with smaller $x$ values). By monotonicity of $f^{\pi}$, all 1 rulings are to its right (with higher $x$ values). Therefore, $x_i$, which is smaller than $x_j$, will be closer to the closest 0 ruling and farther from the closest 1 ruling. Hence, $x_i$ will be ruled as 0 if $x_j$ was.

(3) Consider applications $x_l$ with $\pi^{-1}(j) \leq \pi^{-1}(l) < \pi^{-1}(i)$ (including $x_j$). Those that have been ruled as 0 according to $\pi$ ($l \leq b$) will certainly be ruled as 0 according to $\pi'$, because they are presented after a history that has one additional 0 ruling (and all the others are the same as in $\pi$). There are those, however, that have been ruled as 1 according to $\pi$ ($l > b$), and now they are presented after a history with an additional 0 ($x_i$). However, for each such $l$ we have $x_l > x_j > x_i$. This means that the nearest-neighbor of $x_l$ with a 0 ruling is anyway not $x_i$, and thus the ruling for $x_l$ will remain 1.

(4) Finally, for $l$ with $\pi^{-1}(l) > \pi^{-1}(i)$, each application is presented after the same history according to $\pi$ and to $\pi'$.[§]

We now proceed inductively. If the permutation $\pi^{(1)} = \pi'$ thus defined does not satisfy (i), we apply the same procedure to obtain a new permutation $\pi^{(2)}$ which will also satisfy $\bar{r}^{\pi^{(2)}} = \bar{r}^{\pi}$, and we proceed to generate a sequence of such permutations. The sequence will have to be finite (and end with a permutation that satisfies (i)) because at each step we reduce the number of pairs $(i, j)$ such $i < j \leq b$ and $i, j$ that violate (i).

Finally, we apply a symmetric procedure to $i, j > b$ to generate a permutation that satisfies (ii). Observe that such a procedure does not change the relative locations of $i < j \leq b$, so that if we start with a permutation that satisfies (i) we end with a permutation that satisfies both (i) and (ii). This completes the proof of Lemma 1. □

Back to the proof of Theorem 1, we wish to find out, for a given $b \in \{0, ..., n\}$, if there is a permutation $\pi$ such that $\bar{r}^{\pi} = \bar{r}^{b}$, and to find it if such exists. We wish to prove that the problem can be solved by a polynomial-time algorithm, and the Lemma above simplifies the task: we now know that it suffices to check whether there exists a $b$-monotone $\pi$ such that $\bar{r}^{\pi} = \bar{r}^{b}$. We now turn to describe the algorithm.

We define a binary relation on $(x_i)_{i \leq n}$, denoted $\succ$, and interpreted as "has to appear later than in $\pi$". We will define the (incomplete) relation for some pairs of applications, and the algorithm will check whether the transitive closure of $\succ$, $\succ^*$, might generate a cycle. If this is the case, we will prove that no $\pi$ yields $\bar{r}^{\pi} = \bar{r}^{b}$. Otherwise we will take a transitive and acyclical completion of $\succ^*$ and prove that it defines the permutation we seek.

We start with defining $\succ$ between pairs of applications that are on the same side of $b$:

(i) For every $i < j \leq b$, $j \succ i$ ;

(ii) For every $i > j > b$, $j \succ i$ .

These conditions are not needed for the desired rulings, and are imposed just for our convenience, while Lemma 1 guarantees that the additional conditions will not generate spurious impossibilities.

For $i \leq b < j$ we wish to add to $\succ$ the substantial condition $(i, j)$ (and/) or $(j, i)$ if a correct ruling for one of the applications will necessarily imply an incorrect ruling for the other. To see the intuition, consider the example $H = ((0, 0), (1, 1), (0.4, 0), (0.6, 1))$ discussed above. If $x_1 = 0.4$ is correctly ruled and appears first, history will include $(0.4, 0)$. When $x_2 = 0.6$ is presented, it will have to be ruled at 0 rather than 1. Hence, we conclude that $x_2$ should be presented before $x_1$, or $1 \succ 2$. As we also have $2 \succ 1$ by symmetric reasoning, a cycle was found and the ruling $\bar{r}^{b}$ (for $b = 1$) is impossible.

Generalizing this intuition, we say that, for $i \leq b < j$,

(iii) $j \succ i$ if $x_j - x_i < x_i - x_{i-1}$ (with $x_0 = 0$)

(iv) $i \succ j$ if $x_j - x_i < x_{j+1} - x_j$ (with $x_{n+1} = 1$).

It might be useful to depict $\succ$ by its $n \times n$ incidence matrix $A = A(\succ)$ (with $A_{ij} = 1$ if $i \succ j$ and 0 otherwise). The diagonal consists of 0's. The northwest $b \times b$ matrix has 1's above its diagonal and 0's below it, while the southeast $(n-b) \times (n-b)$ matrix – the other way around. Finally, the actual distances between the $x$'s are reflected by the remaining submatrices. Note that the computation of the matrix $A$ can be done in $O\left(n^2\right)$ steps.

[‡] The number of elements, $\pi^{-1}(j) + 1$, is the sum of the $\pi^{-1}(j) - 1$ elements of $(x_k)$ and the first two "obvious cases" which are assumed at the beginning of every history.

[§] We mention (4) as separate from (1) because it requires the inductive steps in (2) and (3).

The algorithm now proceeds to compute the transitive closure of $\succ$, to be denoted $\succ^*$. It is well-known (and straightforward) that this can be done in $O\left(n^4\right)$ steps. Specifically, consider the Boolean product of incidence matrices, where "Boolean" means $*$ stands for conjunction and $+$ for disjunction. (Thus, addition and multiplication over values $0, 1$ are identical to their algebraic definition apart from the fact that $1 + 1 = 1$). The matrix $A^l$ $(l \geq 1)$ will then be the incidence matrix of $\succ$-paths with length $l$: $A^l_{ij} = 1$ iff there are $l - 1$ indexes, $d_1, ...d_{l-1} \leq n$, such that $i \succ d_1 \succ ... \succ d_{l-1} \succ j$.[¶] For every $i, j$, $i \succ^* j$ iff $\sum_{l=1}^{n} A^l_{ij} = 1$. Thus, the incidence matrix of $\succ^*$ is $A^* = A^*(\succ) = \sum_{l=1}^{n} A^l$. Since the computation of $A^l$ given $A^{l-1}$ (and $A$) can be performed in $O\left(n^3\right)$ steps, $A^*(\succ)\ [= A(\succ^*)]$ can be computed in $O\left(n^4\right)$ steps.

Given $A^*(\succ)$, the algorithm proceeds to check whether $\succ$ has cycles. Specifically, if, for some $i \leq n$, we have $A^*_{ii} = 1$, the algorithm halts and declares $\bar{r}^b$ as infeasible. Otherwise, $\bar{r}^b$ is feasible. To find a permutation $\pi$ such that $\bar{r}^\pi = \bar{r}^b$, the algorithm proceeds as follows. If, for every $i \neq j$, we have $A^*_{ij} + A^*_{ji} = 1$, then $\succ^*$ is a linear order and it defines a permutation $\pi$. (We will shortly argue that for this $\pi$ we have $\bar{r}^\pi = \bar{r}^b$.) If not, we consider a linear order which is an extension of $\succ^*$. Such a linear order exists (this is a special case of Szpilrajn theorem), and can be computed in polynomial time: for a pair $i \neq j$ with $A^*_{ij} = A^*_{ji} = 0$, we can make an arbitrary choice of the ranking between the two. Formally, we fix $i$ and define $\succ' = \succ^* \cup \{(i, j)\}$. Next, we compute $(\succ')^*$ as above. It is straightforward (and, again, well-known) that $\succ'$ has no cycles.[‖] If it so happens that $(\succ')^*$ defines a linear order, we are done, and if not, we proceed in the same way. After no more than $O\left(n^2\right)$ steps (each taking up to $O\left(n^4\right)$) of computation, we end up with a permutation $\pi$ such that $i \succ j$ implies $\pi^{-1}(j) < \pi^{-1}(i)$.

We now wish to show that, (I) if the algorithm computes such a permutation $\pi$, then $\bar{r}^\pi = \bar{r}^b$, and (II) if it ends up with the conclusion that $\succ$ contains a cycles, no such permutation exists. Starting with (I), assume that the algorithm found a permutation $\pi$ that agrees with $\succ$ (so that $i \succ j$ implies $\pi^{-1}(j) < \pi^{-1}(i)$). We need to show that each $i$ is ruled as specified by $\bar{r}^b$, that is, that for $i \leq b$ we have $r^\pi_i = 0$ and for $i > b - r^\pi_i = 1$. The proof is done by induction on $i$ for $i \leq b$, and on $(n - i)$ for $i > b$. Starting with the former, observe first that, thanks for the definition of $\succ$ (part (i)), $\pi^{-1}(l) < \pi^{-1}(i)$ for $l < i$. In particular, $\pi^{-1}(i-1) < \pi^{-1}(i)$. By the induction hypothesis, $r^\pi_{i-1} = 0$. Next, consider $j$ with $j > b$ such that $\pi^{-1}(j) < \pi^{-1}(i)$. Even if the corresponding $x_j$ has been ruled (as we wish it to be) as 1, it is too far from $x_i$ to make the latter be ruled as 1 as well: had it been the case, namely, $x_j - x_i < x_i - x_{i-1}$, by part (iii) of the definition of $\succ$ we would have $j \succ i$ and then $\pi^{-1}(j) > \pi^{-1}(i)$, a contradiction. Hence the closest application to $x_i$ in the history defined by $\pi$ is $x_{i-1}$ and $r^\pi_i = 0$ follows. A symmetric argument implies that $r^\pi_i = 1$ for all $i > b$.

Turning to (II), we observe that a cycle in $\succ$ implies (by parts (iii) and (iv) of the definition of $\succ$) that there is no $b$-monotone permutation $\pi$ with $\bar{r}^\pi = \bar{r}^b$. Lemma 1 implies that in this case there can be no other such permutation $\pi$ either.

This completes the proof of the theorem. □

**Proof of Theorem 2.** We will prove that the corresponding yes/no ("decision") problem is NP-complete. That is, we ask whether, given the data of the problem and a threshold $v$, there exists a permutation $\pi$ whose $U$ value is at least $v$, and prove that this problem is NP-complete. This will imply the statement of the Theorem. Formally,

**Problem 1.** ***OPTIMAL-NN-PERMUTATION:*** *Given $m$, rational $(x_i, u(x_i, 0), u(x_i, 1))_{i \leq n}$ ($x_i \in [0,1]^m$) and a number $v$, is there a permutation $\pi$ such that $U(\bar{r}^\pi) \geq v$?*

We prove this by reduction of the SET-COVER problem to OPTIMAL-NN-PERMUTATION. As a reminder the former is defined by

**Problem 2.** ***SET-COVER:*** *Given $L, W \geq 2$, a set of $W$ subsets of $\mathcal{L} \equiv \{1, ..., L\}$, $\mathfrak{S} = \{S_1, ..., S_W\}$, and $W_0 \leq W$, are there $W_0$ subsets in $\mathfrak{S}$ whose union contains $\mathcal{L}$? (That is, are there indices $1 \leq j_1 < ... < j_{W_0} \leq W$ such that $\bigcup_{l \leq w} S_{j_l} = L$ ?)*

and it is well-known to be NP-complete.

Let there be given an instance of SET-COVER: $L, W \geq 1$, $\mathfrak{S} = \{S_1, ..., S_W\}$ (where $S_j \subset \{1, ..., L\}$ for $j \leq W$) and $W_0 \leq W$. We construct an instance of OPTIMAL-NN-PERMUTATION, $(x_i)_{i \leq n}$, $U = (u(x_i, 0), u(x_i, 1))_{i \leq n}$, as follows. We set $m = L + 2$. There will be four types of applications $x_i$ (the following sets will be pairwise disjoint):

(I) Element-applications: for each $l \leq L$ there will be $x^E_l$;

(II) Set-applications: for each $w \leq W$ there will be $x^S_w$;

(III) Transmitters: there will be $M_T$ applications $x^T_w$;

(IV) Zero-poles: and another $M_Z$ applications $x^P_w$

where $M_T, M_Z$ are integers to be specified below. (We will have to verify that they are bounded by polynomials of the problem.)

The general idea of the construction is the following. Most applications will be located closer to $\tilde{0}$ than to $\tilde{1}$, so that it will be easy to obtain a ruling $r = 0$ for them. However, the desirable payoff of $v$ will only be obtained if each of the element-applications $\left(x^E_l\right)_l$ gets to be ruled as 1. The ruling of 1, which starts at $(1, 1, 1)$, will be transmitted to the element-applications via the set-applications. There will be a cost to each such ruling, so that a high-$U$ permutation will have to be frugal in set-applications that get the ruling 1. Given a "frugal" choice of the sets (i.e., no more than $W_0$ of them), each set will be able to transmit the ruling 1 to each of its elements. This "transmitting" will be obtained by a sequence of applications that are payoff-neutral, and

[¶] This is proved inductively: there is an $l$-long $\succ$-path from $i$ to $j$ iff there exists an index $r \leq k$ such that there exists an $(l-1)$-long $\succ$-path from $i$ to $r$ and $r \succ j$. This is precisely the disjunction of conjunctions performed by Boolean multiplication of incidence matrices.

[‖] If there exists a cycle of $\succ'$, since there were no cycles of $\succ^*$, the cycle must involve the new pair $(i, j)$. This means that there existed a $\succ^*$-path from $j$ to $i$, which also means that there existed a $\succ$- path from $j$ to $i$, contrary to our assumption.

that generate a chain of nearest-neighbors from a given set-application to a given element-application, for any set-element pair where the element belongs to the set. It seems intuitive that, using sufficiently many dimensions, one can generate such a set of applications, and we will indeed prove this below. The basic intuition is that we design a "switchboard", so that wires connect applications and transmit electric current, as it were, from set-applications to element-applications.[**] Thus, if there is a set cover for our problem, we can use it to define a permutation by which all the element-applications get ruled as 1 and no more than $W_0$ of the set-applications get to be ruled as 1.

However, to show the converse direction one needs a more involved construction: we need to be sure that any permutation that rules all element-applications and no more than $W_0$ of the set-applications as 1 indeed defines a cover. Specifically, for every element-application that is ruled as 1 we need to be able to trace back a chain of transmitters that leads to a set-application that was also ruled as 1. This can be difficult to establish as the various transmitters are located in $[0,1]^m$ and they can be rather close to each other in various unplanned ways. To guarantee that such "short-circuits" don't occur, we add the zero-poles. These are additional applications, which will be easily ruled as 0's, and will have to be ruled as 0's in order to obtain the payoff $v$. Each transmitter will have a zero-pole that is sufficiently close to it, but not too close: the transmitter will be the nearest-neighbor of the zero-pole, but the converse will not hold: for the transmitter, the closest neighbors will be the two consecutive transmitters in the chain, and only the third closest one will be the zero-pole. Thus we will be able to prove by induction that, if a given application at the end of the chain (the element-application) was ruled as 1, it must be the case that all the transmitters along the chain are ruled as 1, and the 1-rulings will indeed identify, for each element-application, a set-application such that the set contains the element. We will obviously have to verify that this construction can be done in polynomial time.

With this intuition in mind, we specify the payoff matrix according to the types of applications:

| Type | $u(x_i, 0)$ | $u(x_i, 1)$ |
|---|---|---|
| Element-application | $0$ | $W+1$ |
| Set-application | $0$ | $-1$ |
| Transmitter | $0$ | $0$ |
| Zero-pole | $W+1$ | $0$ |

Thus, there is a cost of 1 for "using" a set-application (that is, having it be ruled as 1), but covering all the elements is more important: missing even one element-application (having it being ruled as 0 instead of 1) is costlier than using all the set-applications. The same would apply to the zero-pole applications, only these have to be ruled as 0 rather than 1. We set

$$v = (L + M_Z)(W+1) - W_0$$

and observe that, for any ruling, $\bar{r}$, we have the desired payoff, $U(\bar{r}) \geq v$, iff all the element-applications get ruled as 1, all zero-pole applications get ruled as 0, and no more than $W_0$ get ruled as 1.

We now turn to describe the location of the applications above. First, we would like to have all interesting applications in a cube that is close to $\tilde{0}$. Specifically, we will focus on the cube defined by the main diagonal $\left\{\tilde{0}, 0.5 \cdot \tilde{1}\right\}$. All set-applications will be located on the "lower plane", in the square defined by the convex hull of $\left\{\bar{0}, 0.5 \cdot \tilde{e}^1, 0.5 \cdot \tilde{e}^2, 0.5 \cdot \left(\tilde{e}^1 + \tilde{e}^2\right)\right\}$ (where $\tilde{e}^j$ denotes the $j$-th unit vector). Thus, all points on the square have 0 in coordinates $3, ..., L+2$. Their exact location is not crucial, but it is important that we can bound the distances between them from below. Specifically, let us locate the $W$ set-applications in an equidistant way along the segment (again, including its extreme points)

$$\left\{ \left(x^1, x^2, 0, ..., 0\right) \;\middle|\; \begin{array}{c} x^1 + x^2 = 0.5 \\ 0 \leq x^1, x^2 \leq 0.5 \end{array} \right\}$$

Thus, the minimal distance between two set-applications is $\frac{\sqrt{2}}{2(W-1)}$.

The element applications will be located on different axes. Specifically, we locate them at $\left\{0.5 \cdot \tilde{e}^{l+2}\right\}_{l=1,...L}$. The distance between any two of these is $\frac{\sqrt{2}}{2}$ and each is closer to $\tilde{0}$ than to $\tilde{1}$. Let $\varepsilon = \frac{\sqrt{2}}{8(W-1)}$ and thus the distance between any two applications of types [(I) and (II)] is larger than $4\varepsilon$.

We now introduce the notion of "wires". We will have two such concepts:

A. An *unprotected wire* is a sequence of transmitters $(x_i)_{i=1}^d$ such that (A1) all $(x_i)_{i=1}^d$ are on a straight line segment (in $[0,1]^3$); and (A2) the distance between two consecutive transmitters is fixed at $\varepsilon$: $\|x_i - x_{i-1}\| = \varepsilon$ for all $1 < i \leq d$.

B. A *protected wire* is a sequence of transmitters and zero-poles $(x_i, z_i)_{i=1}^d$ such that (B1) $(x_i)_{i=1}^d$ is an unprotected wire; (B2) for all $i \leq d$, $\|x_i - z_i\| = \frac{3}{2}\varepsilon$; and (B3) for all $i, j \leq d$, if $i \neq j \leq d$, $\|x_i - z_j\|, \|z_i - z_j\| > \frac{3}{2}\varepsilon$.

It is easy to verify that each wire $(x_i)_{i=1}^d$ can be augmented to a protected wire $(x_i, z_i)_{i=1}^d$: for each $i$, we can find a segment going through $x_i$, that is perpendicular to the segment on which $(x_i)_{i=1}^d$ reside, and locate $z_i$ at a distance $\frac{3}{2}\varepsilon$ from $x_i$ on that segment. To guarantee (B3), we can select perpendiculars (at different $x_i$'s) that are parallel to each other, and locate the zero-poles $z_i$ on different sides of the segment.[††]

[**] In fact, given that the transmission is made by sheer proximity, without a physical connection, one is tempted to think of synaptic connections.

[††] If they are located on the same side, the points $x_i, x_{i-1}, z_i, z_{i-1}$ will be the vertices of a square, and the distance between $z_i$ and $z_{i-1}$ will be equal to $\frac{3}{2}\varepsilon$. This would mean that the nearest neighbor of, say, $z_i$, isn't uniquely defined. If, however, we locate them on different sides, we get $\left\|z_i - z_{i-1}\right\| = \sqrt{10}\varepsilon$.

In the following, when we use wires, we will make sure that property (B3) holds not only for the $x_i$ in the wire. That is, we will verify that, for each zero-pole $z_i$ the corresponding transmitter $x_i$ is the closest application in the entire set of applications.

We will use wires and protected wires to connect the set- and element-applications. We will extend the definitions to situations in which the endpoints of a wire are set-applications or element-applications (rather than transmitters).

We will concatenate (protected or unprotected) wires in the obvious way. Each wire contains transmitters on a straight line segment, and concatenation will allow us to change the direction in which the wire transmits 1 rulings.

We are now equipped to describe the location of the transmitters and zero-poles. In the following, we wish to introduce (protected and unprotected) wires between given points. As the distance between two such points need not be a multiple of $\varepsilon$, we allow each wire to be the concatenation of two such wires.[‡‡] For protected wires, the zero-pole at the intersection of the two wires has to be located in such a way that it is not too close to other $x_i$'s.

We construct four types of wires:

(I) We introduce an unprotected wire going from $\tilde{1} = (1, 1, 1, ..., 1)$ to $\tilde{e}^1 + \tilde{e}^2 = (1, 1, 0, ..., 0)$, and then concatenate it with an unprotected wire going from $\tilde{e}^1 + \tilde{e}^2$ to $0.5 \cdot \left(\tilde{e}^1 + \tilde{e}^2\right)$. This will allow us to have 1 rulings at the latter application, and from there spread into the cube whose main diagonal is $\left\{\tilde{0}, 0.5 \cdot \tilde{1}\right\}$. Notice that no application in this cube can have the ruling 1 before at least some transmitters on this concatenated wire are ruled as 1. This wire is unprotected, because some of its elements are closer to $\tilde{1}$ than to $\tilde{0}$, and zero-poles cannot be located around it without the risk that some of them would be ruled as 1. However, we are not too troubled by short-circuits along this wire, as it will be enough to show that, given a cover, we can use this wire with no short-circuits (that is, we will choose to present its elements in the "right" order and have them all ruled as 1) and, in the converse direction, we will find the at-most-$W_0$ sets that produce a cover before getting to this wire (on our back from the element-applications, as it were).

(II) We introduce a protected wire going from $0.5 \cdot \left(\tilde{e}^1 + \tilde{e}^2\right)$ to each of the set-applications.

(III) In order to connect the set-applications and the element-applications, we introduce a protected wire from each set-application to each element-application for the elements in the set. (Thus, we have an additional $\sum_{w=1}^{W} |S_w|$ wires, each connecting a point $\left(x^1, x^2, 0, ..., 0\right)$ to a point $0.5 \cdot \tilde{e}^{l+2} = (0, 0, 0, ..., 0.5, 0, ..., 0)$).

Before proceeding to prove the validity of the construction, we wish to verify that the numbers of transmitters and zero-poles required, $M_T, M_Z$, are not too large. We proceed by the types of wires introduced above:

(I) The single (unprotected) wire going from $\tilde{1}$ to $\tilde{e}^1 + \tilde{e}^2$, and then to $0.5 \cdot \left(\tilde{e}^1 + \tilde{e}^2\right)$ crosses a distance of $\sqrt{m} + \frac{\sqrt{2}}{2}$. It thus uses no more than $\frac{2\sqrt{m}+\sqrt{2}}{2\varepsilon}$ transmitters.

(II) For each subset $S_w$, we have a protected wire going from $0.5 \cdot \left(\tilde{e}^1 + \tilde{e}^2\right) = (0.5, 0.5, 0, ..., 0)$ to a point $x^1\tilde{e}^1 + x^2\tilde{e}^2 = \left(x^1, x^2, 0, ..., 0\right)$ where $x^1 + x^2 = 0.5$. The maximal distance between these is 0.5 and thus the total number of transmitters, and the total number of corresponding zero-poles, is (each) bounded by $\frac{0.5W}{\varepsilon} = \frac{W}{2\varepsilon}$.

(III) Finally, for each of the $LW$ pairs we may need to have a wire that connects a point $\left(x^1, x^2, 0, ..., 0\right)$ with $x^1 + x^2 = 0.5$ to a point $0.5 \cdot \tilde{e}^{l+2}$. The distance between these is at most $\frac{\sqrt{2}}{2}$. Ranging over all such pairs, we get the bound $\frac{\sqrt{2}LW}{2\varepsilon}$.

Adding up the above, we have

$$\begin{aligned} M_Z &\leq \frac{W}{2\varepsilon} + \frac{\sqrt{2}LW}{2\varepsilon} \\ &\leq \frac{\left(\sqrt{2}L + 1\right)W}{2\varepsilon} \end{aligned}$$

and

$$\begin{aligned} M_T &\leq M_Z + \frac{2\sqrt{m} + \sqrt{2}}{2\varepsilon} \\ &\leq \frac{\left(\sqrt{2}L + 1\right)W}{2\varepsilon} + \frac{2\sqrt{m} + \sqrt{2}}{2\varepsilon} \end{aligned}$$

As $\varepsilon = \frac{\sqrt{2}}{8(W-1)}$, $\frac{1}{2\varepsilon} < 4W$ and we have

$$\begin{aligned} M_Z &\leq 4\left(\sqrt{2}L + 1\right)W^2 \\ M_T &\leq 4\left(\sqrt{2}L + 1\right)W^2 + 8\left(\sqrt{m} + 1\right)W \end{aligned}$$

which means that both are bounded by polynomials of $L, W$. This also proves that the construction of the problem can be done in polynomial time.

We finally turn to prove that the problem we constructed has a permutation $\pi$ such that $U\left(\bar{r}^{\pi}\right) \geq v = \left(L + M_Z\right)(W + 1) - W_0$ iff there is a cover of $\mathcal{L} \equiv \{1, ..., L\}$, using no more than $W_0$ from the collection $\mathfrak{S} = \{S_1, ..., S_W\}$. Assume, first, that such a cover exists. As described in the construction, we choose a permutation $\pi$ with the following properties:

(a) First, $\pi$ lists all the zero-poles. These are all closer to $\tilde{0}$ than to $\tilde{1}$ and are thus ruled as 0's.

[‡‡] Alternatively, one could allow wires to stretch along non-linear curves, as long as the distance between points is monotonically increasing in their index, so that the nearest neighbors of $x_i$ are only $x_{i-1}, x_{i+1}$.

(b) Next, the permutation $\pi$ lists all the set-applications for sets $S_w$ that are not used in the cover, and they are ruled to be 0's, as they are also closer to $\tilde{0}$ than to $\tilde{1}$. This guarantees that these set-applications will not (accidentally, as it were) be ruled as 1 and incur unnecessary cost.

(c) Next, $\pi$ follows the transmitters from $\tilde{1}$ to $(0.5, 0.5, 0, ..., 0)$ (in the order defined by the unprotected wire). Each of these is ruled as 1, because it is closer to its predecessor than to any other application (including the zero-poles that are all within the cube defined by $(0, 0, ..., 0)$ and $(0.5, 0.5, ..., 0.5)$ ).

(d) Following that, the permutation lists all the transmitters in the protected wires leading from $(0.5, 0.5, 0, ..., 0)$ to each of the set-applications for sets $w$ that are used in the cover. Again, following a wire in its intended order results in a sequence of 1 rulings. At this point a cost of (at most) $W_0$ is incurred. (The order by which the wires are used is immaterial. Indeed, the permutation $\pi$ need not complete one wire before starting another.)

(e) For each element $l$ there exists a set $w$ such that $l \in S_w$. For one such $w$, $\pi$ lists the transmitters along the protected wire leading from the set-application to the element-application, and repeats the process for all $l$, where their order is immaterial.

(f) Finally, $\pi$ lists all remaining applications.

It is easy to see that any permutation that satisfies the description above obtains the payoff $v$. First, all zero-poles are ruled as 0's. Next, all element-applications are ruled as 1. These two add $(L + M_Z)(W + 1)$ to the payoff. Of the remaining applications, only the set-applications are not payoff-neutral, and of these $\pi$ ruled as 1 at most $W_0$, so that the overall payoff is at least $(L + M_Z)(W + 1) - W_0$.

We now turn to prove the converse. Suppose that a permutation $\pi$ is obtained, so that $U(\bar{r}^{\pi}) \geq v = (L + M_Z)(W + 1) - W_0$. Clearly, such a permutation has to have all the zero-poles ruled as 0, and all the element-applications ruled as 1. Consider such an element $l$ and the application $x_l$. It has a zero-pole $z_l$ that is at a distance of $\frac{3\varepsilon}{2}$ from it. If $\pi$ places $x_l$ before $z_l$, the latter would be ruled as 1, because $z_l$'s nearest neighbor in the entire collection of applications is $x_l$. As the zero-pole $z_l$ is (correctly) ruled as 0, it has to be the case that it preceded $x_l$ according to $\pi$. This, in turn, implies that, when $x_l$ is introduced, one of its closest neighbors (at distance $\varepsilon$) has already been introduced by $\pi$ and ruled as 1. By construction, it is a transmitter on a protected wire that leads from a set-application corresponding to a set $S_w$ with $l \in S_w$. Next, consider this transmitter. It is ruled as 1, but it, too, has a zero-pole next to it. Its two nearest neighbors are along the wire, and they are the only ones closer to it than is the zero-pole. One of them is $x_l$, which we know appears in $\pi$ only later. It has to be the case that the other nearest neighbor appeared before it and was ruled as 1. The argument proceeds by induction along the protected wire, and we conclude that the set-application of $S_w$ has appeared in $\pi$ and has been ruled as 1. Thus, each $l$ has at least one $w$ such that $l \in S_w$ and $w$ has been ruled as 1 at $\bar{r}^{\pi}$. Given the payoff $(L + M_Z)(W + 1) - W_0$, no more than $W_0$ such sets are needed for the cover. This completes the proof of Theorem 2. □
